# Potential Role of *M. tuberculosis* Specific IFN-γ and IL-2 ELISPOT Assays in Discriminating Children with Active or Latent Tuberculosis

**DOI:** 10.1371/journal.pone.0046041

**Published:** 2012-09-28

**Authors:** Elena Chiappini, Chiara Della Bella, Francesca Bonsignori, Sara Sollai, Amedeo Amedei, Luisa Galli, Elena Niccolai, Gianfranco Del Prete, Mahavir Singh, Mario M. D'Elios, Maurizio de Martino

**Affiliations:** 1 Anna Meyer University Hospital, Department of Science for Woman and Child Health, University of Florence, Florence, Italy; 2 Department of Internal Medicine, University of Florence, Florence, Italy; 3 Lionex GmbH, Braunschweig, Germany; Fundació Institut d'Investigació en Ciències de la Salut Germans Trias i Pujol. Universitat Autònoma de Barcelona. CIBERES, Spain

## Abstract

**Background:**

Although currently available IGRA have been reported to be promising markers for TB infection, they cannot distinguish active tuberculosis (TB) from latent infection (LTBI).

**Objective:**

Children with LTBI, active TB disease or uninfected were prospectively evaluated by an in-house ELISPOT assay in order to investigate possible immunological markers for a differential diagnosis between LTBI and active TB.

**Methods:**

Children at risk for TB infection prospectively enrolled in our infectious disease unit were evaluated by in-house IFN-γ and IL-2 based ELISPOT assays using a panel of *Mycobacterium tuberculosis* antigens.

**Results:**

Twenty-nine children were classified as uninfected, 21 as LTBI and 25 as active TB cases (including 5 definite and 20 probable cases). Significantly higher IFN-γ ELISPOT responses were observed in infected *vs.* uninfected children for ESAT-6 (p<0.0001), CFP-10 (p<0.0001), TB 10.3 (p = 0.003), and AlaDH (p = 0.001), while differences were not significant considering Ag85B (p = 0.063), PstS1 (p = 0.512), and HspX (16 kDa) (p = 0.139). IL-2 ELISPOT assay responses were different for ESAT-6 (p<0.0001), CFP-10 (p<0.0001), TB 10.3 (p<0.0001), HspX (16 kDa) (p<0.0001), PstS1 (p<0.0001) and AlaDH (p = 0.001); but not for Ag85B (p = 0.063). Comparing results between children with LTBI and those with TB disease differences were significant for IFN-γ ELISPOT only for AlaDH antigen (p = 0.021) and for IL-2 ELISPOT assay for AlaDH (p<0.0001) and TB 10.3 antigen (p = 0.043). ROC analyses demonstrated sensitivity of 100% and specificity of 81% of AlaDH-IL-2 ELISPOT assay in discriminating between latent and active TB using a cut off of 12.5 SCF per million PBMCs.

**Conclusion:**

Our data suggest that IL-2 based ELISPOT with AlaDH antigen may be of help in discriminating children with active from those with latent TB.

## Introduction

Substantial advances have been recently achieved in the immunological diagnosis of *Mycobacterium tuberculosis* infection [1]. Three *Mycobacterium tuberculosis* specific interferon-γ (INF-γ) release assays (IGRAs) are now commercially available. With respect of tuberculin skin test (TST), IGRAs have a number of advantages: they are minimally influenced by previous bacille Calmette-Guérin (BCG) vaccination or infection by non-tuberculosis mycobacteria, do not cause booster effect, do not necessitate of a double access to health care facility, and interpretation of results is not operator-dependent [2]. In adults IGRAs have been reported to be more specific and at least as sensitive as TST [3], and are currently included in diagnostic algorithms in adult guidelines [4]. However, reported IGRAs sensitivity and specificity largely vary among studies in paediatric populations and caution is recommended regarding their use in children [1], [5], [6], [7]. Moreover, IGRAs do not distinguish LTBI from active tuberculosis (TB) patients [8]. This issue is fundamental for paediatricians since a definite diagnosis of active TB is rare in children. Paediatric TB disease is typically paucibacillary and most TB diagnoses are “probable “diseases, based on TST/IGRA results, clinical symptoms and signs, radiological findings, epidemiological data, and response to antitubercular therapy [1], [5]–[8]. Therefore a test able to distinguish accurately LTBI from active TB would be valuable. IGRAs based on cytokines other than IFN- **γ** or exploring immune response to other mycobacterial antigens besides those included in the commercially available IGRAs are currently under investigations [8].

Previous observations suggest a possible role of IL-2- based ELISPOT assay in addition to the IFN-gamma-based assay in discriminating active from latent TB, as only cells from individuals with LTBI and not those from individuals with active TB has been found to secrete IL-2 after specific stimulation [9]. These results probably reflect the increased number of IL-2 secreting and IL-2/IFN-gamma secreting central memory T cells and the reduced number of IFN-gamma effector T-cells in LTBI patients, associated with the low bacterial replication and low antigen load [10]. In the present study we prospectively evaluated the performances of in-house IFN-γ and IL-2 based ELISPOT assays by the use of a panel of *Mycobacterium tuberculosis* antigens in children consecutively referred to one paediatric infectious disease unit and at risk for TB infection with the aim of investigating possible immunological markers for a differential diagnosis between LTBI and active TB.

## Materials and Methods

### Study subjects

Children at risk for TB infection consecutively referred to our Infectious Disease Unit were prospectively enrolled between 1 st January 2009 and 31^st^ April 2010. Study children were those with clinical suspicion of TB disease and/or in close contact with recently diagnosed cases of contagious TB disease and/or internationally adopted or recently immigrated children coming from countries with a high-prevalence of TB. Children with congenital or acquired immunodeficiency disorders (based on their medical history, clinical examination and/or laboratory tests) were excluded from the study. This study was approved by the ethical committee of the Anna Meyer University Hospital, Florence, Italy. All the parents/tutors of the study children gave written consent to the study.

### Tuberculin skin test (TST)

TST was administered by trained nurses dedicated to our Infectious Disease Unit and was performed according to the Mantoux method by injecting intradermally 5 tuberculin units (in 0.1 mL) of purified protein derivative (Statens Serum Institute, Copenhagen, Denmark) into the volar surface of the forearm. The transversed skin induration was recorded (in mm) after 48–72 hours directly by a pediatrician of the Infectious Disease Unit. Following the American Academy of Pediatrics guidelines [7], a positive TST was defined as an induration size ≥5 mm for children in close contact with known or suspected contagious case of TB disease or for children suspected to have TB disease (based on clinical evidence and/or chest radiograph) and ≥10 mm for children born in countries with a high prevalence of TB and recently immigrated.

### QuantiFERON Gold In tube test (QFT-G-IT)

The QFT-IT assay (Cellestis Inc., Australia) was performed according to the manufacturer's instructions as described previously. After subtracting the value from the negative control, the result was positive if, the antigen-dependent response was ≥0.35 IU, negative if the mitogen–induced response was ≥0.5 IU/mL and the antigen-dependent response was <0.35 IU/mL, and indeterminate if both mitogen-induced and antigen-dependent responses were below cut-off or mitogen-induced response >8 IU/mL.

### ELISPOT Assay

In-house ELIPOT assay detecting IFN-gamma and IL-2 responses were performed using recombinant *M. tuberculosis* antigens (ESAT-6, CFP-10, TB 10.3, AlaDH, Ag85B, PstS1, HspX (16 kDa) protein), provided by Lionex Diagnostics & Therapeutics GmbH (Braunschweig, Germany), as previously reported [10], [11]. Characteristics of the used antigens are summarized in Table 1. Briefly, peripheral blood mononuclear cells (PBMCs; 1×10^6^ cells/mL) of each patients were stimulated with *M. tuberculosis* recombinant antigen (5 µg/mL), and seeded in triplicate in 96-well plates coated with anti–IFN-γ or anti–IL-2 antibody. Cells stimulated with medium alone served as negative controls. Cells stimulated with phytohemoagglutinin (PHA) served as positive controls. IFN-γ and IL-2 ELISPOT microplates were then incubated at 37°C in 5% CO_2_ for 24 hours. At the end of the culture period, plates were washed and incubated for 3 hours with the appropriate biotinylated anti–IFN-γ or anti–IL-2 monoclonal antibody. Streptavidin-HRP complex was then added for 2 hours, followed by the substrate solution. Spot forming colonies (SFCs) were counted using an automated ELISPOT reader (Autoimmune Diagnostika GmbH) and results expressed as number of SFCs per million PBMCs, as described [12]. Laboratory workers were blind to the clinical status of participants.

**Table 1:** Characteristics of the used antigens: all the used antigens were recombinant proteins (from Hoghart et al. [14], modified).

Protein	Gene Bank Accession number	Remarks	Putative function
ESAT-6	Rv3875	6 kDa early secreted antigen	Unknown
CFP-10	Rv3874	10 kDa culture filtrate antigen of *M. tuberculosis*	Unknown
Ag85B	Rv1886c	Secreted antigen, member of the immunodominant complex Antigen 85	Cell wall synthesis, fibronectin binding
PstS1 (38 kDa)	Rv0934	Periplasmic phosphate binding protein	Protein mediated phosphate transport
TB10.3	Rv3019c	Secreted ESAT6-like protein	Unknown
HspX (16 kDa;Acr)	Rv2031c	Heat shock protein. It has been identified as a major protein expressed in dormant state	Stress protein induced by anoxia. It has a role in manteinance of long-term viability during latent asymptomatic infection and a proposed role in replication during initial infection
AlaDH	Rv2780	Secreted L-alanine deydrogenase of *M. tuberculosis*	May play a role in cell wall synthesis (as L-alanine in an important constituent of the peptidoglycan layer) *;* crystal structure varies in the phase of active disease with respect to the state of latent infection in “open” and “closed” ternary forms

### Study design

Information regarding demographic data, prior TB exposure, and past medical history was obtained from each child's parents or tutors or from medical documentation and recorded into the study database. Children were considered vaccinated with BCG whether a clear documentation was available and/or a scar was present. All children underwent clinical evaluation, TST and venipuncture for IGRAs (QFT-G-IT and in-house ELISPOT assay) and results entered into a databse. Blood was taken during the first examination after the parent's or tutor's informed consent had been obtained and before starting any anti-tubercular treatment. Chest radiography was performed in all symptomatic children, in those with a positive TST, and in all contacts aged less than 5 years [7]. Children with suspected pulmonary TB had three sputum or early morning gastric aspirates samples collected for *Mycobacterium tuberculosis* detection (by means of microscopy, polymerase chain reaction and culture) [7]. Chest computed tomography (CT) scan was performed in selected cases at the paediatrician's discretion. No suspected or ascertained extra-pulmonary TB cases presented at the Infectious Disease Unit during the study period.

The study received approval from the Ethical Committee of Anna Meyer Children University Hospital.

### Definition of study groups

Study children were classified as not-infected, LTBI cases, or active TB disease cases, following the American Academy Guidelines definition [7]. In the event of discordant TST/QFT-G-IT results, children were assigned to the corresponding group on the bases on the TST result [7]. In particular, asymptomatic children with negative TST were defined as uninfected. LTBI diagnosis was assigned to any child with a positive TST and no clinical or radiographic evidence of active TB [7]. Cases of active TB were defined according to two categories: 1) definite TB, children with *Mycobacterium tuberculosis* cultured or detected by microscopy or molecular methods from sputum or gastric aspirate culture; 2) probable TB: absence of microbiological confirmation but presence of all of the following criteria: (A) clinical symptoms and signs of active TB, (B) abnormal radiography and/or CT scan consistent with lung TB, (C) response to TB therapy plus, (D) either a history of TB contact or travel to a TB-endemic country within the last 24 months [13]. No suspected or ascertained extra-pulmonary TB cases presented at the Infectious Disease Unit during the study period.

## Statistical Analysis

Categorical data were compared using the Chi-squared test (or Fisher's exact test, when expected cell sizes were smaller than five). The Wilcoxon-Mann-Whitney test was used for continuous measurements to test relationships in unpaired analysis, when assumed that the dependent variable is a not normally distributed interval variable. Test concordance was assessed by Cohen's κ-statistics with agreement considered slight'for k≤0.2, ‘fair’ for 0.2<k≤0.4, ‘moderate’for 0.4<k≤0.6, ‘substantial’for 0.6<k≤0.8 and ‘optimal’for 0.8<k≤1.0. Receiver operating characteristic (ROC) curve analysis was conducted to determine the best IL-2 and IFN-γ ELISPOT result thresholds in discriminating between children with active or latent TB, relatively to a specific *M. tuberculosis* antigen, and correspondent sensitivity and specificity were reported. The area under the ROC curve (AUC) and 95% confidence interval (CI) were also calculated. Statistical analysis was performed using the statistical software SPSS for Windows, version 14.0. *P*<0.05 was considered statistically significant.

## Results

Seventy five children were included in the study. Twenty-none children were classified as uninfected, 21 as LTBI and 25 as active TB cases (including 5 definite and 20 probable cases). Characteristics of the study children are summarized in Table 2.

**Table 2:** Characteristic of the 75 study children, according to their final diagnosis.

	Uninfected	Latent TB	Probable TB disease	Definite TB disease
	n = 29	n = 21	n = 20	n = 5
	n (%)	n (%)	n (%)	n (%)
**Age**, months (median and IQR)	77 (28–101)	86 (61–101)	52(27–82)	47 (8–97)
**Immigrated**	21 (72.4)	15 (71.4)	18 (90)	4 (80.0)
**BCG vaccinated**	5 (17.2)	7 (33.3)	1 (5.0)	0 (0.0)
**TST** (mm)				
<5	28 (96.5)	0 (0.0)	0 (0.0)	1 (20.0)
≥5 and <10	1 (3.5)	1 (4.8)	2 (10.0)	0 (0.0)
≥10 and <15	0 (0.0)	5 (23.1)	1 (5.0)	1 (20.0)
≥15	0 (0.0)	15 (71.5)	17 (85.0)	3 (60.0)
**QFT-G-IT result**				
negative	29 (100.0)	5 (23.1)	3 (15.0)	5 (100.0)
positive	0 (0.0)	15 (71.5)	17 (85.0)	0 (0.0)
indeterminate	0 (0.0)	1 (4.8)	0 (0.0)	0 (0.0)

Note. TB: tuberculosis; BCG: Bacille Calmette-Guérin vaccine; TST = tuberculin skin test; QFT-G-IT: QuantiFERON-Gold In Tube test.

Discordant TST/QFT-G-IT result was obtained in four children (one child with definite TB, positive QFT-G-IT and negative TST and three children with probable TB, negative QFT-G-IT and positive TST). The overall agreement between QFT-G-IT and the TST was substantial with a *k* value of 0.679.

### IFN-γ and IL-2 ELISpot results

**Figure 1:**
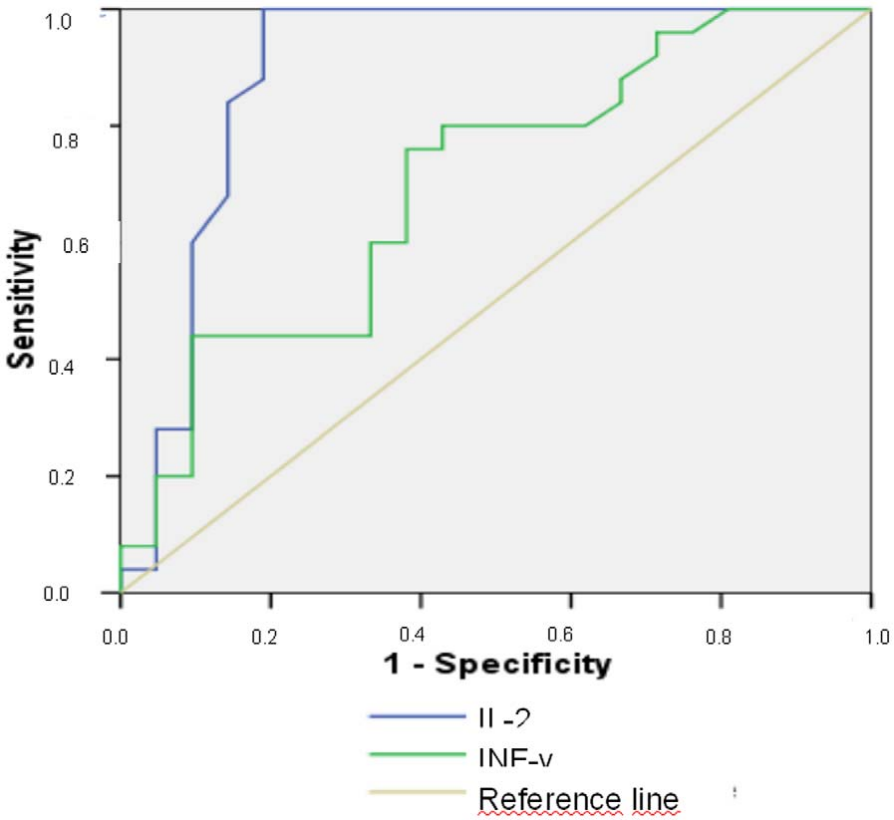
A receiver operator characteristic (ROC) plot is shown, illustrating sensitivity and specificity of AlaDH IFN-γ and IL-2 ELISpot results in discriminating children with latent (n = 21) and overt (n = 25) tuberculosis. Area under the ROC curve was 0.700 (95%IC: 0.547-0.853; p = 0.021 *vs.* the identity - diagonal - line) considering IFN-γ ELISpot and 0,896 (95%IC: 0.785–1.008; p<0.0001 *vs.* the identity - diagonal - line) for IL-2 ELISPOT.

ELISPOT assay results are summarized in Table 3. Significantly higher median values were evidenced in infected *vs.* uninfected children considering IFN-γ ELISPOT responses to ESAT-6 (p<0.0001), CFP-10 (p<0.0001), TB 10.3 (p = 0.003), and AlaDH (p = 0.001), while differences were not significant considering Ag85B (p = 0.063), PstS1 (p = 0.512), and HspX (16 kDa) protein (p = 0.139). Considering IL-2 ELISPOT results significantly different responses were evidenced for ESAT-6 (p<0.0001), CFP-10 (p<0.0001), TB 10.3 (p<0.0001), HspX (16 kDa) (p<0.0001), PstS1 (p<0.0001) and AlaDH (p = 0.001); but not for Ag85B (p = 0.063). Comparing results between children with LTBI and those with TB disease (probable plus definite disease) differences were significant for IFN-γ ELISPOTt only for AlaDH antigen (p = 0.021) (Table 3). With regard to IL-2 ELISPOT assay, significant differences were observed for AlaDH (p<0.0001) and TB 10.3 antigen (p = 0.043), while no difference was evidenced considering other antigens. ROC analyses demonstrated sensitivity of 100% and specificity of 81%, in discriminating between latent and active TB, considering response to AlaDH by IL-2 ELISPOT assay for a cut off of 12.5 SCF per million PBMCs. For IFN-γ ELISPOT assay the performance was much poorer: for the best threshold of 42.0 SCF per million PBMCs sensitivity was 88% and specificity 56% (Figure 1).

**Table 3:** ELISPOT assay results from 75 study children according to final diagnosis.

	Uninfected	Latent TB	Probable TB disease	Definite TB disease	P	P
	(n = 29)	(n = 21)	n = 20	n = 5	Uninfected vs. infected children	Latent vs. overt disease
**IFN-γ ELISpot result** *						
- ESAT-6	35 (1–102)	320 (157–520)	292 (170–631)	330 (190–1575)	<0.0001	0.724
- CFP-10	40 (1–120)	305 (130–625)	280 (76–986)	875 (200–3532)	<0.0001	0.691
- Ag85B	65 (12–152)	150 (47–322)	107 (46–192)	90 (27–1517)	0.063	0.724
- TB 10.3	100 (27–292)	270 (135–465)	252 (80–395)	570 (225–1742)	0.003	0.487
- AlaDH	35 (12.5–110)	85 (17–180)	147 (71–303)	280 (115–1565)	<0.001	0.021
- PstS1	40 (1–122)	50 (1–197)	60 (11–241)	95 (18–982)	0.0512	0.504
- HspX - 16KD	10 (1–162)	85 (1–300)	42 (1–307)	40 (13–1345)	0.139	0.720
**IL-2 ELISpot result** *						
- ESAT-6	10 (1–35)	220 (35–410)	110 (60–402)	420 (155–1365)	<0.0001	0.791
- CFP-10	10 (1–20)	210 (85–710)	160 (100–637)	510 (355–1735)	<0.0001	0.574
- Ag85B	20 (10–75)	40 (10–140)	45 (17–195)	200 (135–1485)	0.063	0.188
- TB 10.3	1 (1–30)	50 (12.5–210)	130 (52–402)	390 (220–1695)	<0.0001	0.043
- AlaDH	1 (1–20)	1 (1–10)	75 (36–130)	340 (110–1365)	0.001	<0.0001
- PstS1	10 (1–50)	20 (1–75)	20 (10–95)	10 (5–155)	<0.0001	0.755
- HspX- 16 KD	1 (1–10)	60 (20–280)	45 (1–155)	240 (45–920)	<0.0001	0.626

Note.

* Results are expressed as median and interquartile range of spot forming colonies per million PBMCs; TB:tuberculosis.

## Discussion

In the present study we evaluated the performances of IFN-γ and IL-2 based ELISPOT assays in children at risk for TB using an array of *M. tuberculosis* specific antigens. We observed significantly different responses between infected and not-infected children with respect to antigens included in the commercially available IGRAs (ESAT-6 and CFP-10), as well as to other mycobacterial antigens (TB 10.3, AlaDH for IFN-gamma and IL-2; HspX and PstS1 only for IL-2 based ELISPOT), but not for Ag86B. This latter result was in contrast to what we expected, considering that Ag85B has been described to elicit a robust immune response in TB patients and it is a candidate protein to be included in new antitubercular vaccines [14]. Indeed Ag85B is one of the most dominant protein antigens secreted from all mycobacteria species, showing extensive cross-reactivity between different species [15]. Thus, exposure to enviromental mycobacteria might sustain an immune response to this antigen in children not-infected by *M. tuberculosis*.

No difference in responses to ESAT-6, CFP-10 and other mycobacterial antigens (Ag85B, PstS1, HpsX) was found between children with latent and active tuberculosis, while significant differences were found in responses to TB 10.3 and AlaDH antigens. TB 10.3 is a member of the large 23 protein ESAT-6 gene family and contains several unique T-cell epitopes strongly recognised by TB patients [13]. The recognition of a number of unique epitopes on TB10.3 suggests that this protein is highly expressed by the bacteria during active TB infection, potentially explaining our finding [16]. To our knowledge this is the first report on a potential role of TB 10.3 to differentiate between LTBI and active TB, deserving further investigations.

Considering AlaDH antigen, the differences observed in children with LTBI and active TB may have a biological explanation. Differently from the other antigens included in our study, AlaDH is the only whose conformation is modified in latent with respect to active TB [17]. It has been demonstrated that AlaDH shows altered expression profile upon adaptation to dormancy: a condition that may be related to the state of latent infection. Though an obligatory aerobic organism, *M. tuberculosis* is able to adapt to and survive in the hypoxic and hostile environment of host macrophages undergoing a dramatic change in gene transcription during a latent infection [8]. AlaDH is an enzyme involved in nitrogen metabolism and has been implicated in the adaptation of mycobacteria to the anaerobic dormant state. The crystal structure varies in the phase of active disease with respect to the state of latent infection in “open” and “closed” ternary forms, thus explaining a possible different host immune response according to the infectious status [17]. We observed that different responses between children with LTBI and active TB were more pronounced with the IL-2 based than with the IFN-γ based ELISPOT. ROC analyses demonstrated that, for a cut off of 12,5 SCF per million PBMC, the sensitivity in discriminating children with LTBI and active TB reached 100% while specificity was 81% by the use of IL-2 based ELISpot assay. The performance of the IFN-γ ELISpot assay was poor, especially due to its low specificity. These findings suggest that IL-2 based ELISPOT assay with AlaDH antigen may be of help in discriminating children with active from those with latent TB. The finding that the differences in response to AlaDH were more pronounced considering IL-2 instead of IFN-γ based ELISPOT assays are not clearly explainable. In contrast with our findings, in adults studies, ESAT-6 and CFP-10 IFN-γ ELISPOT assays were more likely to be positive in recently infected patients, while IL-2 a positive ELISPOT was related to ancient exposure, since this test better explores the central memory T-cell response [9], [18]. A higher production of IL-2 in latently infected people after 72 h incubation has been documented. Thus a prolonged incubation period seems to be essential to reveal increased number of central memory T-cells in LTBI adults [9]. However, it should be remembered that the T_H_1 response may be immature and peculiar in children. In this population IL-2 based IGRAs have been reported to show higher performance than IFN-γ based assays, explaining, at least partially, our results [19]. Further pediatric studies prolonging the culture up to 3 days could provide further information in children. Further pediatric studies prolonging the culture up to 3 days could provide further information in children. We did not observe any difference between children with active or latent diseases when exploring response to HspX (16 kDa) antigen. This antigen has been depicted as one of the most prominent latency proteins [20], and a strong response to HspX, largely restricted to latently infected individuals, has been previously reported by Demissie and colleagues in adults [20]. Discrepancies between our and previous findings at this regard may be due to the limited datasets, or to actual differences in the immune response between adult and paediatric populations.

Our study has several limitations. Definition of study groups was based on TST results and not on QFT-G-IT results according to the most recent US guidelines in children [7]. However, children could have been classified according to their QFT-G-IT instead of TST results and three of our study children with discordant TST+/QFT-G-IT would have assigned to a different group. We repeated the analyses after excluding these children and results were substantially unchanged. Moreover, similarly to the majority of paediatric studies most of the overt TB cases were probable and not definite diseases and in general our dataset is small. Thus even if intriguing, our findings should be confirmed in larger paediatric populations.

### Conclusion

Our data suggest that IL-2 and IFN-gamma based ELISPOT with AlaDH antigen may be of help in discriminating children with active from those with latent TB.
